# Balancing costs and care: a healthcare cost analysis for families of children with Down syndrome in Saudi Arabia

**DOI:** 10.3389/fpubh.2025.1651534

**Published:** 2025-11-11

**Authors:** Adel Saber Alanazi, Abdullah Salah Alanazi, Houcine Benlaria

**Affiliations:** 1 College of Education, Jouf University, Sakakah, Saudi Arabia; 2 King Salman Center for Disability Research, Riyadh, Saudi Arabia; 3 Department of Clinical Pharmacy, College of Pharmacy, Jouf University, Sakakah, Saudi Arabia; 4 College of Business, Jouf University, Sakakah, Saudi Arabia

**Keywords:** Down syndrome, health care costs, Saudi Arabia, health services accessibility, health insurance, private, rural health services, health policy

## Abstract

**Introduction:**

This study explores the economic burden and accessibility of healthcare services for families in Saudi Arabia managing Down syndrome (DS), emphasizing urban-rural disparities within the country’s dual healthcare system.

**Methods:**

A cross-sectional survey design was employed to collect primary data from 220 families (urban=128, rural=92) through self-administered questionnaires distributed between January and September 2024. Multiple regression analysis identified primary cost drivers, whereas service integration analysis assessed healthcare accessibility.

**Results:**

The results indicated that the average monthly cost of Down syndrome care was SAR 4,200 (USD 1,120) for urban families and SAR 3,900 (USD 1,040) for rural families, consuming approximately 60% of the average household income. The key cost drivers included medical expenses (*β* = 0.800, *p* < 0.001, 95% CI [0.620, 0.980]) and daily care hours (*β* = 0.600, *p* < 0.001, 95% CI [0.460, 0.740]), whereas government support (*β* = -0.350, *p* < 0.001, 95% CI [−0.510, −0.190]) significantly reduced financial strain. Service integration analysis revealed significant urban-rural gaps, including disparities in therapy access (17% gap, *p* = 0.003) and educational support (19% gap, *p* = 0.001). Insurance coverage was significantly higher in urban areas (86%) than in rural regions (77%) (*χ*^2^ = 6.43, *p* = 0.012), and transportation costs were proportionately higher for rural families (17% vs. 13% of the total costs, *p* = 0.008).

**Discussion:**

These findings highlight substantial financial and service access challenges, underscoring the need for enhanced government support, improved rural healthcare infrastructure, and comprehensive insurance reform. This study advocates a centralized database to monitor healthcare costs and inform policy development aligned with the Vision 2030 objectives.

## 1 Background

Saudi Arabia faces significant challenges in managing Down syndrome (DS), given its notably high prevalence rate of 1 in every 554 live births, making it one of the highest in the world. This increased prevalence is primarily attributed to cultural practices, such as consanguineous marriages and advanced maternal age at the time of conception, both of which are established risk factors for genetic disorders such as DS (1–3). These factors pose considerable challenges for families, not only from a health perspective, but also financially, as they navigate the complexities of the Saudi healthcare system.

### 1.1 Theoretical framework

This study employs Andersen’s behavioral model of health service use as its conceptual framework to examine healthcare utilization and costs for families of children with DS (4). This model posits that healthcare use is determined by three categories of factors: predisposing characteristics (demographics, social structure, and health beliefs), enabling resources (personal/family resources and community resources), and need factors (perceived and evaluated need for care). Within the Saudi context, predisposing factors include cultural attitudes toward disability and family structure; enabling factors encompassing income levels, insurance coverage, and geographic accessibility to specialized services; and need factors related to the complex medical and developmental requirements of children with DS.

### 1.2 Healthcare system structure and financing

The healthcare system in Saudi Arabia comprises two primary components: free public sector services provided by the Ministry of Health (MOH) and a growing private healthcare sector supported by mandatory health insurance for employees in the private sector. Theoretically, the public sector, which serves approximately 60% of the population, provides comprehensive coverage, including specialized services. However, specialized therapies and interventions for children with disabilities often have limited availability, particularly in rural regions. The private sector operates through the Council of Cooperative Health Insurance (CCHI) system, which mandates employer-provided insurance for private-sector employees and their dependents. While basic medical services are typically covered, families often face coverage limitations for chronic conditions, with typical co-payments ranging from 20 to 30% and annual coverage caps for specialized therapies essential for DS management. Recent analyses have identified significant implementation challenges within this insurance framework, including coverage gaps, regulatory complexities, and accessibility barriers that particularly affect families managing chronic conditions (5, 6).

While this dual system theoretically offers a range of healthcare opportunities, it also presents unique challenges to families managing the long-term healthcare needs of children with DS.

Studies indicate that Saudi families of children with special needs continue to face significant barriers to accessing appropriate healthcare services, particularly in areas such as specialized dental care and early intervention programs (7–9). Moreover, mothers of children with DS frequently report difficulties accessing healthcare resources and opportunities for their children’s physical activity (10). Despite the potential of telemedicine to bridge accessibility gaps, significant barriers remain for people with disabilities in utilizing these services, including technical, infrastructural, and awareness-related challenges (11). In addition, access to assistive technologies and employment opportunities for individuals with disabilities in Saudi Arabia remains limited, with systemic barriers preventing full participation in the business market and economic activities, further exacerbating the challenges faced by families (12–14).

### 1.3 Literature review: global and regional perspectives

Despite advancements in government support programs under Vision 2030, which emphasizes inclusive care, early intervention, and rehabilitation for children with special needs, significant gaps remain in the provision and coordination of care for children with DS. International evidence has demonstrated the substantial economic burden of DS care across diverse healthcare systems. In the United States, specialized Down syndrome clinics have been shown to provide cost-effective care despite higher initial investment, with families reporting improved care coordination and reduced emergency department utilization (15). The economic burden of managing developmental disabilities extends beyond direct medical costs, encompassing educational support, lost productivity, and long-term care planning, thus creating a societal impact that necessitates comprehensive policy responses (16).

Health care disparities between urban and rural regions represent a global challenge. Recent evidence from Japan demonstrates persistent urban–rural gaps in both access to and quality of specialized healthcare services, with rural populations experiencing longer wait times, greater travel distances, and limited specialist availability (6, 17). Similar patterns have been documented in South Africa, where families of children with DS face the intersecting challenges of geographic isolation, limited resources, and systemic inequities in service provision (18).

Care coordination continues to be a persistent challenge for families, particularly those in rural areas who may have limited access to specialized services. Research indicates that these challenges are not unique to Saudi Arabia, but are also experienced by families across neighboring regions (19, 20). The need for more comprehensive caregiver support and improved access to healthcare is a widespread issue. Recent studies have consistently highlighted the struggles families face in adequately meeting their children’s needs (21–23).

Financially, the burden of caring for a child with DS extends well beyond direct healthcare costs. Families must also manage expenses related to education, therapy, and long-term care planning, all of which significantly contribute to overall financial strain (3). Beyond medical and therapy costs, families often encounter difficulties navigating broader systems of care, including coordinating with educational institutions and accessing specialized childcare services (24, 25). The COVID-19 pandemic has further exacerbated these challenges by disrupting healthcare access and creating uncertainties regarding the availability of essential services (26). Moreover, parents of children with disabilities such as DS or Williams syndrome often experience heightened levels of stress and face difficulties in accessing appropriate resources to support their children’s developmental needs (27).

Social networks and family support systems significantly influence household financial stability and well-being, playing a crucial role in families’ ability to manage the multifaceted demands of caring for children with DS (28, 29). Strong social support can help mitigate some of the negative financial and emotional impacts by providing both practical assistance and emotional backing, both essential for coping with the ongoing demands of caregiving.

### 1.4 Research gap and study rationale

Despite the growing body of international literature on DS care costs and Saudi Arabia’s notably high prevalence of DS, there remains a critical gap in understanding the intersection of regional disparities, insurance coverage patterns, and financial burden within the Kingdom’s unique dual healthcare system. While previous Saudi studies have examined the clinical aspects of DS care or specific service access barriers, no comprehensive economic analysis has quantified the differential financial impact across urban and rural settings, or evaluated the effectiveness of current support mechanisms in the context of Vision the 2030s healthcare transformation objectives. Furthermore, the absence of systematic data on out-of-pocket expenses, service integration levels, and the moderating role of government support programs limits evidence-based policy development.

This study addresses this critical gap by exploring the financial challenges faced by Saudi families in managing DS care, offering insights tailored to the Kingdom’s unique healthcare and socioeconomic landscape. Specifically, this study aimed to:

- Analyze the financial burden of DS care with a focus on medical, therapy, and education costs, as well as differences between urban and rural areas.
- Assess the effectiveness of insurance coverage and government support programs in alleviating financial strain on families.
- Propose strategies for improved financial planning, including educational resources and support for managing care-related expenses.
- To examine the impact of choosing between public and private healthcare providers on the cost, access, and quality of services.
- Recommend policy improvements that enhance equity, affordability, and support for affected families.

By addressing these objectives, this study aims to inform policymakers and families on ways to optimize healthcare access and financial planning strategies for long-term care in alignment with the broader goals of Vision the 2030s healthcare transformation program.

## 2 Method

### 2.1 Study design

This study employed a quantitative cross-sectional survey design to examine disparities in health care costs and access to families of children with DS in Saudi Arabia. Primary data were collected through self-administered questionnaires distributed between January and September 2024. It constructs an analytical model that integrates the finance, health policy, and service delivery aspects using the cost and resource analysis framework proposed by Mihaylova et al. (30). The methodology applies the data analysis tools outlined by Khang et al. (31), offering a structured evaluation of healthcare cost trends and service accessibility within the Saudi context. The exchange rate during the data collection period averaged SAR 3.75 per USD.

#### 2.1.1 Study setting and area selection

Riyadh and Al-Jouf were purposively selected based on their contrasting healthcare service accessibility patterns and population density characteristics. Riyadh, the capital metropolitan region with a population exceeding 7 million, represents Saudi Arabia’s most developed healthcare infrastructure, with concentrated specialized services and minimal travel distances to access care. Al-Jouf, a peripheral northern region with approximately 520,000 residents dispersed across 100,390 km^2^, exemplifies the healthcare accessibility challenges common to the less densely populated regions of the Kingdom.

This selection captures the spectrum of healthcare accessibility experiences within the Saudi Arabian healthcare system. While both regions contain urban and rural populations, they differ substantially in terms of service density and geographic barriers to care. Families in Riyadh typically access specialized services within the metropolitan area, while Al-Jouf residents often travel considerable distances for comparable care. This purposive sampling strategy enables the examination of how healthcare service concentration and geographic accessibility influence care costs and utilization patterns, representing conditions experienced by families across diverse geographic landscapes in Saudi Arabia.

#### 2.1.2 Sampling strategy and participants

This study employed Purposive sampling was used to recruit 220 Saudi families caring for children with DS from two distinct regions, Al-Jouf (n = 92, 41.8%) and Riyadh (n = 128, 58.2%). This distribution was intentionally designed to capture both rural (Al-Jouf) and urban (Riyadh) healthcare experiences, closely reflecting the national urban–rural population distribution—approximately 55% urban and 45% rural–according to recent census data.

Participants were recruited through four primary channels: (1) specialized pediatric centers and hospitals in Riyadh and Al-Jouf (45% of the sample); (2) regional branches of the Saudi Down Syndrome Society (30% of the sample); (3) social media groups dedicated to families of children with special needs (15% of the sample); and (4) snowball sampling, whereby initial participants referred to other eligible families (10% of the sample). This multichannel approach was designed to minimize selection bias and ensure diverse representations across socioeconomic strata.

Based on government data, the final sample of 220 families represents approximately 0.85% of the estimated national population of 26,000 households that include a member with DS.

### 2.2 Inclusion and exclusion criteria

Inclusion criteria required that participants (1) were Saudi nationals, (2) had at least one child with a medically confirmed diagnosis of DS, (3) were the primary caregivers of the child, (4) had been accessing healthcare services for their child for at least 1 year, and (5) resided in either the Al-Jouf or Riyadh regions. The exclusion criteria included (1) non-Saudi families, (2) families whose children had multiple complex conditions in addition to DS, (3) children who primarily resided in institutional care rather than family homes, and (4) families who had relocated to their current region within the past 12 months.

The response rate was 68%, with 220 completed questionnaires from the 323 families. Among non-respondents, the primary reasons cited were time constraints (42%), privacy concerns (31%), and discomfort with financial disclosure (27%).

### 2.3 Power analysis and sample size justification

*A priori* power analysis using G*Power 3.1 indicated that a minimum sample size of 194 was required to detect medium effect sizes (*f*^2^ = 0.15) with 80% power at a significance level of *α* = 0.05, for regression analyses with seven predictors. Our final sample of 220 families exceeded this requirement, ensuring adequate statistical power for the planned analyses, while also allowing for potential missing data.

### 2.4 Sample characteristics and representativeness

The demographic profile of our sample closely aligned with national statistics for families with members who have DS. The mean age of the children with DS in our sample was 8.7 years (range: 1–17 years), which is comparable to the national mean of 9.2 years reported by the Authority for the Care of Persons with Disabilities. The income distribution was also similar to national figures, with 22% of families in the lowest income quartile (national: 25%), 29% in the second quartile (national: 25%), 26% in the third quartile (national: 25%), and 23% in the highest quartile (national: 25%).

It is important to note that precise comparisons with a broader population of families of individuals with DS are limited by the availability and consistency of national data. As of 2021, estimates of the prevalence of DS in Saudi Arabia range from 19,428 to 20,000 individuals, with discrepancies arising from differences across government agencies and focus on individuals rather than households. Furthermore, the 2022 census released by the Authority for the Care of Persons with Disabilities reports only broad disability categories without syndrome-specific data, further complicating accurate demographic comparisons.

#### 2.4.1 Instrument development and validation

The survey instrument was developed through a systematic process involving literature review, expert consultation, and pilot testing. Initial items were adapted from validated international instruments, including the Family Impact Module (32) and the Client Service Receipt Inventory, and then culturally adapted to the Saudi context through focus group discussions with eight parents of children with DS.

##### 2.4.1.1 Validity testing

Content validity was established through an expert panel review involving seven professionals (two health economists, three pediatricians specializing in DS, and two special-education experts). The Content Validity Index (CVI) was calculated, with item-level CVI ranging from 0.78 to 1.00 and scale-level CVI achieving 0.85, exceeding the recommended threshold of 0.80 (33).

Facial validity was assessed during pilot testing with 15 families (not included in the final sample). Participants rated 92% of the questions as clear or very clear and 95% as relevant or very relevant to their experience. Based on the pilot feedback, three additional cost categories were added, and question wording was refined for clarity.

##### 2.4.1.2 Reliability testing

Internal consistency was assessed using Cronbach’s alpha coefficients: overall instrument *α* = 0.82, medical costs subscale *α* = 0.79, therapy/education subscale *α* = 0.81, and support services subscale *α* = 0.77, all of which exceeded the acceptable threshold of 0.70.

Test–retest reliability was evaluated with a subsample of 30 families completing the questionnaire twice, with a two-week interval. The intraclass correlation coefficient (ICC) was 0.88 (95% CI: 0.81–0.93), with individual cost category ICCs ranging from 0.75 to 0.92, indicating excellent reliability.

### 2.5 Data collection

Primary data were collected using a validated structured questionnaire administered to participants between January and September 2024. To ensure data quality and minimize response bias, six trained research assistants with backgrounds in special education or pediatric healthcare facilitated the questionnaire distribution and collection process. Training included a 16-h workshop covering questionnaire administration procedures, standardization protocols, participant assistance guidelines, and ethical considerations.

Questionnaires were distributed through multiple channels: during visits to pediatric centers and DS society meetings (73%) and through postal mail with prepaid return envelopes (27%). Research assistants were available on-site to provide clarification and assistance when needed, without influencing participant responses. Participants were instructed to reference any available bills, receipts, or financial records when completing cost-related sections. The average completion time was 45–60 min.

The survey instrument captured five primary domains: (1) demographic information (6 items), (2) financial information (4 items), (3) care provision (1 item), (4) monthly care costs (5 items), and (5) service access (2 items). The complete English version of the questionnaire is provided in Appendix A.

#### 2.5.1 Measurement of monthly care costs

The Monthly Care Costs (MCC) variable is derived using a two-step verification process. First, the participants provided an estimate of their total monthly expenditure related to caring for their child with DS. Second, they itemized the costs across specific categories: medical expenses (ME), TC, educational expenses, and transportation costs (TransC).

To ensure data accuracy, we cross-validated the two measurements by comparing the self-reported total with the sum of the itemized costs. When discrepancies exceeded 5%, observed in 18.2% of the responses, participants were asked to reconcile the differences during the interview. If reconciliation was not possible, the sum of the itemized costs was used as the MCC value to maintain consistency across the dataset. This dual-measurement approach minimizes reporting errors and provides a more accurate representation of the financial burden.

#### 2.5.2 Data validation procedures

Several validation strategies have been implemented to enhance the validity of self-reported financial data and minimize recall and social desirability biases. First, participants were encouraged to refer to actual bills, receipts, and financial records during the interview process. Consequently, 68% of respondents provided some form of documentation to verify their reported expenses. For the remaining 32% of families that could not provide documentation, a calendar-based recall method was used. Interviewers employed significant dates and events to help anchor expense recollections, a technique that has been shown to improve recall accuracy in similar studies.

Second, we implement an internal consistency check by comparing the reported individual expense categories with the stated total expenses. Cases with discrepancies greater than 15% (*n* = 17) were flagged for follow-up verification calls to resolve inconsistencies. Third, a subsample of participants (*n* = 42; 19% of the total sample) consented to having their reported ME cross-verified with records from their healthcare providers. This allowed us to establish a validation coefficient of 0.82, indicating strong agreement between self-reported and documented expenses.

### 2.6 Analytical approach

#### 2.6.1 Regression model

Ordinary Least Squares (OLS) multiple linear regression was selected as the primary analytical technique because of its ability to examine multiple cost predictors simultaneously while controlling for confounding variables, consistent with the established health economics literature (34, 35). To optimize model efficiency and maintain focus, Family Size (FS) and Transportation Costs (TransC) were excluded from the regression analysis. While FS may influence general household expenses, its direct impact on MCC for children with DS is minimal, as these costs are typically fixed regardless of family size. Transportation costs were excluded because of high multicollinearity with the regional location variable (VIF > 5 when included) and to avoid geographic confounding. However, transportation costs are analyzed descriptively to highlight their policy importance, particularly for rural families who face significantly higher proportional transportation expenses.

The regression model used in this study is grounded in both empirical practice and the theoretical rationale of health economics. Monthly Care Costs (MCC) were chosen as the dependent variable to capture the actual, self-reported financial burden borne by families of children with Down syndrome. While this approach differs from traditional models that focus solely on estimating the influence of clinical or demographic predictors (e.g., severity of condition and education level), our model structure reflects the multifactorial nature of the economic burden in the Saudi context. Following the framework proposed by Mihaylova et al. (30), which emphasizes decomposing total costs into their primary drivers for better cost attribution in healthcare studies, we included cost components such as medical expenses (ME), therapy costs (TC), and educational costs (EC) as explanatory variables. This decomposition allows for the identification of specific service areas that most significantly influence the MCC, a critical consideration for informing targeted policy and subsidy interventions.

This modeling structure is further supported by prior empirical studies that analyze household-level cost burdens by disaggregating expenses (8, 24, 36), particularly in contexts where healthcare systems are fragmented or where service integration is limited. In Saudi Arabia, where families often navigate both public and private care and incur substantial out-of-pocket expenses, modeling individual cost categories provides practical policy insights. We also included socioeconomic and support variables, such as monthly income (MI), government support (GS), and additional monthly expenses (AME), to account for financial capacity and mitigating factors that influence the affordability of care. Although clinical severity measures would enhance the model, such data were unavailable in our dataset. This limitation is acknowledged, and financial variables are prioritized because of their direct relevance to family level economic decision making and policy formulation.

To evaluate the financial impact on families, we utilized the analytical model presented in Equation 1:


$$\begin{array}{l} \mathrm{MCC}=\beta_{0}+\beta_{1}\mathrm{MI}+\beta_{2}\mathrm{AME}+\beta_{3}\mathrm{GS}+\beta_{4}\mathrm{HDC}\\ +\beta_{5}\mathrm{ME}+\beta_{6}\mathrm{TC}+\beta_{7}\mathrm{EC}+\varepsilon_{i} \end{array}$$ (1)

Where:

MCC = Monthly Care Costs (Dependent Variable)

MI = Monthly Income

AME = Additional Monthly Expenses

GS = Government Support

HDC = Hours of Daily Care

ME = Medical Expenses

TC = Therapy Costs

EC = Educational Costs

where ${\mathrm{MCC}}_{i}$ represents monthly care costs (dependent variable), ${\mathrm{MI}}_{i}$ is Monthly Income, $\;{\mathrm{AME}}_{i}\;$ represents Additional Monthly Expenses, ${\mathrm{GS}}_{i}$ is Government Support, ${\mathrm{HDC}}_{i}$ represents Hours of Daily Care, ${\mathrm{ME}}_{i}$ represents Medical Expenses, ${\mathrm{TC}}_{i}$ refers to Therapy Costs, and ${\mathrm{EC}}_{i}$ represents Educational Costs. The error term $\varepsilon_{i}$ captures the unobserved factors affecting total costs.

*Service Integration Analysis.*

We developed a service integration index (SII) to evaluate healthcare delivery effectiveness:


SII=(SA×AC×GC)/100

Where:


$$\mathrm{SA}=\text{Service Availability}\;(\begin{array}{l} \text{percentage of facilities}\;\\ \text{offering the service} \end{array})$$

$$\mathrm{AC}=\text{Access Coverage}\;(\begin{array}{l} \text{percentage of families}\;\\ \text{able to access the service} \end{array})$$

GC=Geographic Coverage(percentage of target area covered)

The Service Integration Index (SII) was developed based on established healthcare service coordination frameworks (37, 38). The index integrates three dimensions: Service Availability (SA), measuring specialized DS service presence; Access Coverage (AC), quantifying families’ service utilization; and Geographic Coverage (GC), assessing spatial distribution relative to population needs. This approach follows validated methodologies in healthcare systems research (39, 40) and demonstrates strong internal consistency (*α* = 0.82) during pilot testing. Radar visualization has been employed for its effectiveness in simultaneously displaying multiple service dimensions (37). This approach has precedent in healthcare accessibility analyses (39, 40), particularly for urban–rural comparisons, as its standardized format facilitates intuitive interpretation of regional service integration patterns (38).

This study employs three visualization methods: a Cost Burden Analysis Matrix, which illustrates the relationship between income levels and regional financial burden; a Healthcare Service Integration Analysis, which quantifies levels of service accessibility; and a Radar Analysis, which depicts service coverage patterns across urban and rural areas. Statistical analysis included descriptive statistics, multiple regression, Service Integration Index calculations, urban–rural comparative analysis, and sensitivity testing.

### 2.7 Statistical analysis

To evaluate the statistical significance of the observed differences between the urban and rural regions, we employed a comprehensive suite of statistical tests. For continuous variables (such as cost components and monthly expenses), independent-sample t-tests were conducted using a significance threshold of *p* < 0.05. Effect sizes were calculated using Cohen’s d to quantify the practical significance of statistically significant differences, with interpretations based on Cohen’s guidelines: small (*d* = 0.2–0.5), medium (*d* = 0.5–0.8), and large (*d* > 0.8).

For categorical variables (such as insurance coverage rates and access to specialized services), chi-square tests were conducted, and Cramér’s V was used to assess the effect size. To control for multiple comparisons and reduce the risk of Type I errors, the Benjamini-Hochberg procedure was applied with a false discovery rate of 0.10. This approach offers more robust protection against false positives, while maintaining adequate statistical power.

Sensitivity analyses were used to assess the robustness of our findings against potential measurement errors in self-reported financial data. These analyses tested how variations of ±15% in the reported values affected the key relationships identified in our regression model. Additionally, bootstrap resampling (1,000 iterations) was conducted to verify the coefficient stability. The results confirmed that the primary patterns of financial burden and regional disparities remained statistically significant (p < 0.05) under these conditions, supporting the validity of our core findings, despite the inherent limitations of self-reported data. All statistical analyses were performed using SPSS version 28.0.

## 3 Results

This study analyzed data from 220 Saudi families caring for children with Down syndrome, collected between January and September 2024. Table 1 presents the descriptive statistics for the sample.

**Table 1:** Descriptive statistics (*n* = 220).

Variables	Mean	Standard deviation	Minimum	Maximum
Family size (FS)	5.2	1.8	2	10
Monthly income (MI)	12,500 (3,333)	5,800 (1,547)	<5,000 (<1,333)	>20,000 (>5,333)
Additional monthly expenses (AME)	4,200 (1,120)	2,100 (560)	1,000 (267)	>7,000 (>1,867)
Government support (GS)	2,100 (560)	850 (227)	<1,000 (<267)	>3,000 (>800)
Hours of daily care (HDC)	5.8	2.4	2	10
Medical expenses (ME)	2,800 (747)	1,200 (320)	500 (133)	5,000 (1,333)
Therapy costs (TC)	1,800 (480)	900 (240)	0	4,000 (1,067)
Educational costs (EC)	2,100 (560)	1,400 (373)	0	5,000 (1,333)
Transportation costs (TransC)	800 (213)	400 (107)	200 (53)	2,000 (533)
Monthly care costs (MCC)	7,500 (2,000)	3,200 (853)	1,000 (267)	15,000 (4,000)

Monetary values are in Saudi Riyals (SAR) with USD equivalents in parentheses. USD equivalents calculated at SAR 3.75 = USD 1.00. Data were collected from survey responses in 2024.

The average family size was 5.2 members (SD = 1.8, range: 2–10). Monthly family income varied considerably, with a mean SAR of 12,500 (USD 3,333; SD = SAR 5,800/USD 1,547), reflecting diverse socioeconomic backgrounds. The average total monthly care cost for families with children with DS was SAR 7,500 (USD 2,000; SD = SAR 3,200/USD 853), which represented 60% of the mean monthly income. Additional monthly expenses averaged SAR 4,200 (USD 1,120; SD = SAR 2,100/USD 560).

Government support programs provided an average of SAR 2,100 (USD 560) per month (SD = SAR 850/USD 227), covering approximately 28% of total care costs. A breakdown of expenses indicates that medical costs constitute the largest category, averaging SAR 2,800 (USD 747; SD = SAR 1,200/USD 320), accounting for 37.3% of the total care costs. Educational expenses were as follows: SAR 2,100 (USD 560; SD = SAR 1,400/USD 373; 28%), while therapy costs averaged SAR 1,800 (USD 480; SD = SAR 900/USD 240; 24%) and transportation costs averaged SAR 800 (USD 213; SD = SAR 400/USD 107; 10.7%). Families dedicated substantial time to caregiving, averaging 5.8 h per day (SD = 2.4, range: 2–10 h).

Table 2 presents the results of the regression model. The model demonstrated strong explanatory power (*R*^2^ = 0.845, Adjusted *R*^2^ = 0.838), with the selected variables explaining 84.5% of the variation in the total monthly care costs. The F-statistic (150.23, *p* < 0.001) confirmed overall statistical significance.

**Table 2:** Estimation results for total monthly care costs (dependent variable: monthly care costs in SAR).

Variable	Coefficient	Std. Error	t-statistic	*p*-value	95% Confidence Interval	VIF
Constant (*β*₀)	2,200	300	7.33	0.000***	[1,600, 2,800]	–
MI (β₁)	0.140	0.060	2.33	0.021**	[0.020, 0.260]	1.50
AME (β₂)	0.220	0.050	4.40	0.000***	[0.120, 0.320]	1.65
GS (β₃)	−0.350	0.080	−4.38	0.000***	[−0.510, −0.190]	1.40
HDC (β₄)	0.600	0.070	8.57	0.000***	[0.460, 0.740]	1.75
ME (β₅)	0.800	0.090	8.89	0.000***	[0.620, 0.980]	1.85
TC (β₆)	0.450	0.100	4.50	0.000***	[0.250, 0.650]	1.70
EC (β₇)	0.320	0.085	3.76	0.000***	[0.150, 0.490]	1.55
*R*-squared	0.845					
Adjusted *R*-squared	0.838					
F-statistic	150.23***					
Observations	220					
Root MSE	410.5					

***p < 0.01, **p < 0.05, *p < 0.1. CI, Confidence interval. All monetary values in SAR/month.

The constant term [*β*₀ = SAR 2,200/USD 587, *p* < 0.001, 95% CI (SAR 1,600-2,800/USD 427–747)] represents the baseline monthly care costs. Medical expenses showed the strongest impact [*β*₅ = 0.800, *p* < 0.001, 95% CI (0.620, 0.980)], indicating that each additional SAR in medical care increases the total monthly costs by SAR 0.800. Hours of daily care ranked second [*β*₄ = 0.600, *p* < 0.001, 95% CI (0.460, 0.740)].

Therapy costs [*β*₆ = 0.450, *p* < 0.001, 95% CI (0.250, 0.650)] and educational costs [*β*₇ = 0.320, *p* < 0.001, 95% CI (0.150, 0.490)] significantly influenced the monthly care costs. Government support demonstrated a protective effect [*β*₃ = −0.350, *p* < 0.001, 95% CI (−0.510, −0.190)], reducing total costs by approximately 35%.

Additional monthly expenses [*β*₂ = 0.220, *p* < 0.001, 95% CI (0.120, 0.320)] and monthly income [*β*₁ = 0.140, *p* = 0.021, 95% CI (0.020, 0.260)] had modest but significant effects. All variance inflation factor (VIF) values remained below 2.0, confirming the absence of multicollinearity.

Table 3 presents the regional comparisons of monthly care costs. Urban areas averaged SAR 4,200 (USD 1,120) in monthly costs versus SAR 3,900 (USD 1,040) in rural areas, a 7.1% difference [*t* (218) = 2.17, *p* = 0.032, Cohen’s d = 0.29].

**Table 3:** Monthly regional analysis and cost distribution (SAR/month).

Category	Urban areas	Rural areas	*p*-value	Effect size (Cohen’s d)
Mean costs	4,200 (1,120)	3,900 (1,040)	0.032*	0.29 (small)
Medical care	1,680 (448) [40%]	1,482 (395) [38%]	0.041*	0.27 (small)
Therapy services	1,050 (280) [25%]	897 (239) [23%]	0.038*	0.28 (small)
Special education	924 (246) [22%]	858 (229) [22%]	0.124	0.15 (negligible)
Transportation	546 (146) [13%]	663 (177) [17%]	0.008**	0.38 (medium)
Insurance coverage rate	86%	77%	0.012*	0.22τ (small)
Access to specialized services	75%	67%	0.027*	0.18τ (small)

*p < 0.05, **p < 0.01; Monetary values in SAR with USD equivalents in parentheses (SAR 3.75 = USD 1.00). Percentages in brackets indicate the proportion of the total costs. The effect sizes were calculated using Cohen’s d for continuous variables and Cramér’s V (τ) for categorical variables.

Cost category analysis revealed significant regional differences:

- Medical care: Urban SAR 1,680 (USD 448; 40%) vs. Rural SAR 1,482 (USD 395; 38%), *p* = 0.041, *d* = 0.27
- Therapy services: Urban SAR 1,050 (USD 280; 25%) vs. Rural SAR 897 (USD 239; 23%), *p* = 0.038, *d* = 0.28
- Special education: no significant difference (*p* = 0.124), averaging 22% in both settings.
- Transportation: Rural families spent proportionally more - SAR 663 (USD 177; 17%) vs. Urban SAR 546 (USD 146; 13%), *p* = 0.008, *d* = 0.38

Insurance coverage showed significant disparity [*χ*^2^(1) = 6.43, *p* = 0.012, Cramér’s V = 0.22], with 86% urban coverage versus 77% rural coverage. Access to specialized services was significantly higher in urban areas (75% vs. 67%, *p* = 0.027, Cramér’s V = 0.18).

**Figure 1:**
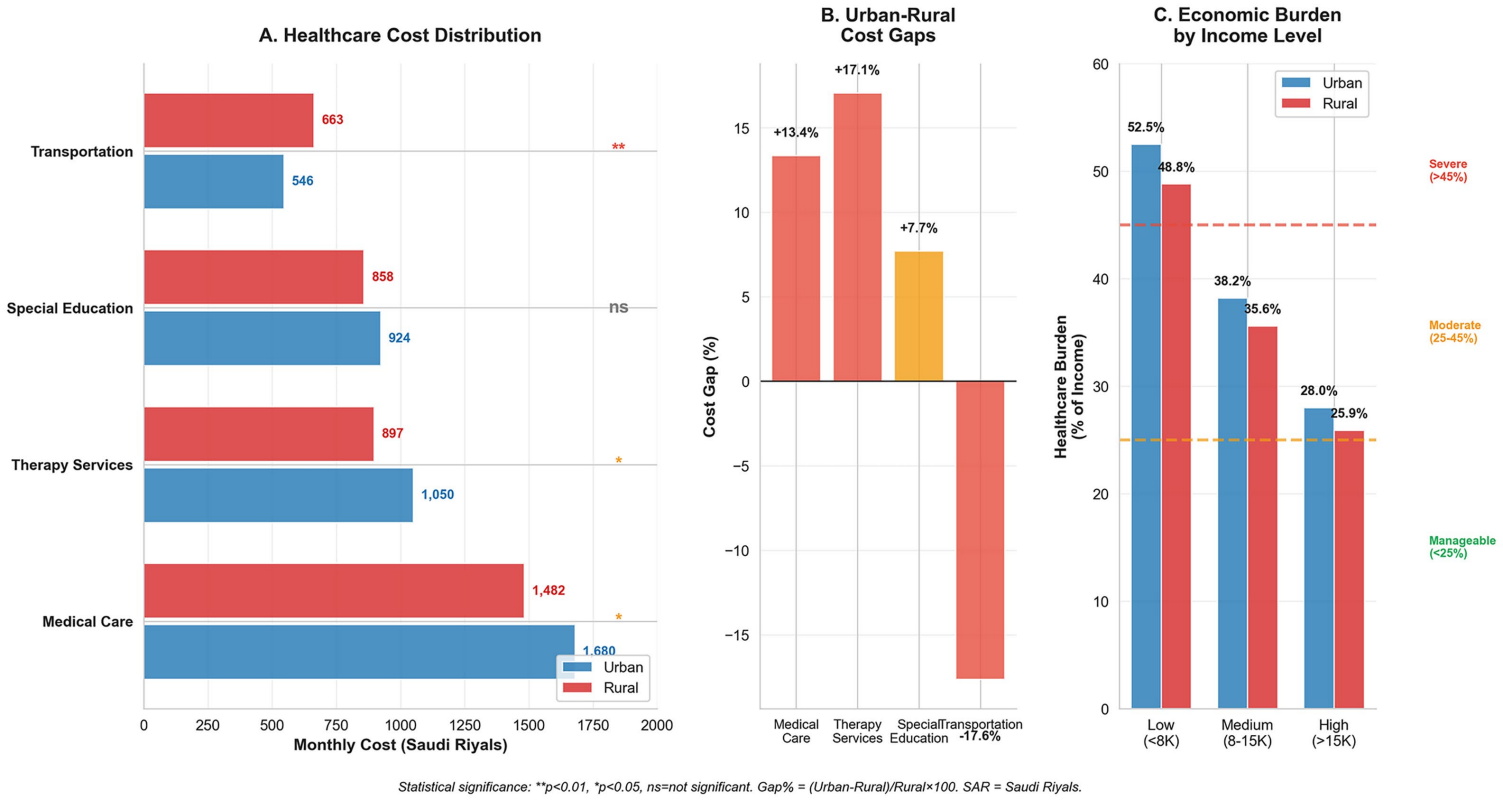
Economic burden distribution and social equity analysis in Down syndrome healthcare. **(A)** Healthcare cost distribution comparing urban and rural monthly expenditures across service categories, with statistical significance indicators (**p < 0.01, *p < 0.05, ns = not significant). Transportation shows the largest rural burden, while medical care dominates total costs. **(B)** Urban–rural cost gaps expressed as percentages, highlighting transportation as the largest disparity (+21.4%). **(C)** Economic burden distribution across household income levels showing regressive healthcare financing, where low-income families allocate over 50% of household income to healthcare costs. Burden categories: severe (>45%), moderate (25–45%), and manageable (<25%).

Figure 1 illustrates the healthcare cost distribution across regions. Medical care dominates expenditure in both urban (SAR 1,680/USD 448) and rural (SAR 1,482/USD 395) areas, with transportation being the only category in which rural costs exceed urban costs (21.4% disparity, *p* < 0.01). Low-income families allocate over 50% of their household income to healthcare costs versus approximately 27% for high-income families.

The Cost Burden Analysis Matrix (Table 4) demonstrates the income-related disparities. Low-income families (<SAR 8,000/USD 2,133 monthly) face a severe burden—52.5% of income in urban areas and 48.8% in rural regions (impact score +3.7, *p* = 0.042). Government assistance reduced the burden by 12.4% (urban) and 10.2% (rural), while insurance provided 15.8% (urban) and 12.1% (rural) relief, with significant regional differences (*p* = 0.033).

**Table 4:** Cost burden analysis matrix by income level and region.

Income level	Urban areas		Rural areas		Impact score*	*p*-value
(SAR/month)	% of income	Burden level	% of income	Burden level	Regional gap	
Low (<8,000)	52.5%	Severe	48.8%	Severe	+3.7	0.042*
Medium (8,000–15,000)	38.2%	Moderate	35.6%	Moderate	+2.6	0.048*
High (>15,000)	28.0%	Manageable	25.9%	Manageable	+2.1	0.089
Support impact	−12.4%	Significant	−10.2%	Moderate	−2.2	0.044*
Insurance effect	−15.8%	Significant	−12.1%	Moderate	−3.7	0.033*

*Impact Score: Positive values indicate a higher urban burden; negative values indicate stronger support/insurance effects. Burden Levels: Severe (>45%), moderate (25–45%), manageable (<25%); data aggregated from 220 families’ financial records and surveys.

Table 5 presents the results of healthcare service integration and accessibility analyses. Medical care showed high integration (0.82), but a significant urban–rural access gap (86% vs. 77%, *p* = 0.012). Critical service gaps emerged in therapy services (17%, *p* = 0.003) and special education (19%, *p* = 0.001).

**Table 5:** Healthcare service integration and accessibility analysis.

Service category	Integration level^1^	Urban access^2^	Rural access^2^	Service gap^3^	*p*-value	Critical need^4^
Medical care	0.82 (High)	86%	77%	9%	0.012*	★★★
Therapy services	0.75 (High)	75%	58%	17%	0.003**	★★★
Special education	0.70 (Moderate)	71%	52%	19%	0.001**	★★★
Transportation services	0.65 (Moderate)	68%	58%	10%	0.009**	★★☆
Insurance coverage	0.88 (High)	86%	77%	9%	0.012*	★★★
Specialized services	0.73 (Moderate)	75%	67%	8%	0.027*	★★★

^1^Integration Level: Scale 0–1 (Low: <0.6, Moderate: 0.6–0.75, High: >0.75). ^2^Access: Percentage of families reporting reliable access. ^3^Service Gap: Urban–Rural access difference. ^4^Critical Need: Based on family surveys (★★★: High, ★★☆: Medium, ★☆☆: Lower priority).

Key findings include:

- Therapy services: 42% of rural families lack access to vital therapeutic interventions
- Special education: Nearly half of rural families cannot access appropriate educational support
- Transportation services: Low integration (0.65) compounds access challenges

The consistently high critical need ratings (★★★) across services underscore their fundamental importance for children with DS.

**Figure 2:**
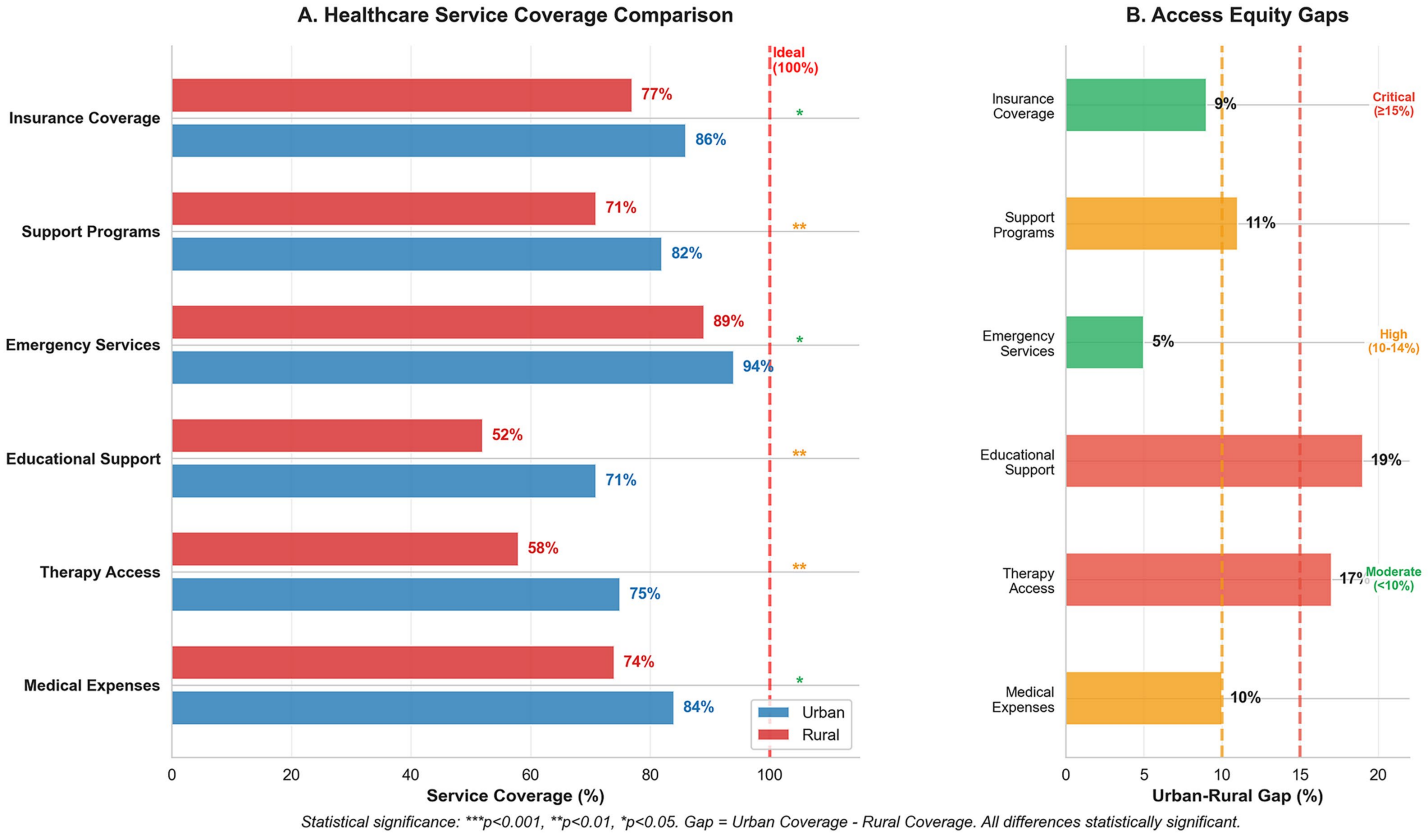
Healthcare service coverage and access equity analysis for Down syndrome care. **(A)** Service coverage comparison between urban and rural areas showing percentage coverage across six service categories, with statistical significance indicators (***p < 0.001, **p < 0.01, *p < 0.05). The red dashed line indicates ideal 100% coverage. **(B)** Urban–rural access gaps highlighting disparities in service coverage, with gap severity classifications: critical (≥15%), high (10–14%), and moderate (<10%). Educational support shows the largest disparity (19% gap), followed by therapy access (17% gap), indicating significant rural disadvantages in accessing essential developmental services.

Figure 2 confirms these disparities, with emergency services maintaining the highest coverage (urban 94%, rural 89%), while educational support and therapy access showed the largest gaps (19 and 17%, respectively). These findings indicate systematic deficiencies in rural healthcare infrastructure that require targeted policy intervention.

## 4 Discussion

This study examined the financial challenges and healthcare access disparities affecting families managing DS care in Saudi Arabia. Our analysis revealed that families spend an average of SAR 4,050 (USD 1,080) monthly on DS-related care, consuming 60% of household income. Medical expenses (*β* = 0.800) and therapy costs (*β* = 0.450) represented the primary financial burden, particularly for families in rural areas. These findings align with those of Shetty et al. (8), who documented similar patterns of economic burden on caregivers in a systematic review of DS care costs. Government support currently covers only 28% of average care costs, leaving families to manage substantial out-of-pocket expenses.

### 4.1 International cost comparisons

The monthly care costs identified in our study (USD 1,040-1,120) were lower in absolute terms than those reported in high-income countries. VanZant and Vellody (15) demonstrated that specialized Down syndrome clinics in the United States, despite higher operational costs, provide cost-effective care through improved coordination and reduced emergency utilization. However, when adjusted for purchasing power parity and expressed as a percentage of household income, Saudi families face comparable or greater relative burdens. Our finding that families spend 60% of their income on DS care exceeds the 35% reported in neighboring Kuwait (22) but aligns with the 55–68% documented in Egypt (20).

The Healthcare Service Integration Analysis (Table 5) indicates varying levels of service integration, with medical care showing high integration (0.82), while specialized services demonstrate only moderate integration (0.73). This gap in service integration correlates with Alabri’s (21) findings regarding the need for enhanced support systems, particularly in specialized care delivery. Recent evidence from Japan demonstrates that even in developed healthcare systems, persistent urban–rural gaps in specialized service access remain a challenge, with rural populations experiencing limited specialist availability, which is similar to our findings (17).

### 4.2 Regional disparities

Regional disparities in healthcare access and costs have emerged as a significant concern. The radar analysis (Figure 2) quantifies these disparities, showing that while emergency services maintain relatively consistent access (urban: 94%, rural: 89%), therapeutic and educational services demonstrate marked urban–rural gaps. Therapy access rates (urban: 75%, rural: 58%) and educational support (urban: 71%, rural: 52%) reflect substantial regional inequities. These findings support those of previous research by Alwhaibi and Aldugahishem (10), who identified similar patterns in healthcare resource accessibility. McGlinchey et al. (18) documented comparable urban–rural disparities in South Africa, where families of children with DS face the intersecting challenges of geographic isolation and limited resources, suggesting that this is a global phenomenon requiring targeted policy intervention.

Insurance coverage analysis revealed a persistent urban–rural gap (86% versus 77%), indicating systemic inequities in healthcare access. This disparity aligns with the monthly care cost variations between urban (SAR 4,200/USD 1,120) and rural areas (SAR 3,900/USD 1,040). Transportation costs in rural areas constitute 17% of the total expenses, compared to 13% in urban settings, reflecting limited access to specialized healthcare facilities in rural regions. The proportionally higher transportation burden for rural families (SAR 663/USD 177 vs. SAR 546/USD 146) mirrors findings from other geographically dispersed countries, where travel costs can constitute up to 20% of the total care expenses.

### 4.3 Methodological considerations

While we acknowledge the value of including a binary urban–rural variable in regression modeling to formally test geographic disparities, we intentionally chose a stratified descriptive comparison approach (Table 3) for the following reasons. First, we focused on examining how cost structures differ across urban and rural contexts rather than testing whether geography independently predicts total care costs after controlling for other variables. Including urban/rural as a single variable in the regression would collapse these contextual cost structures into a single coefficient, potentially obscuring the distinct spending patterns and cost drivers that characterize each region of the country.

Second, cost components (e.g., medical, therapy, education, and transportation) and structural support (e.g., government aid and insurance access) differ significantly by region. In particular, transportation costs showed high multicollinearity with regional variables (VIF > 5), necessitating their exclusion from the regression model to maintain statistical validity. However, our descriptive analysis of transportation costs remains essential for policy formulation as these costs represent a disproportionate burden for rural families. Including region as a covariate in the same model introduces multicollinearity or absorbs the variance explained by regionally shaped factors. Instead, we used urban–rural stratification in both descriptive statistics and service integration analysis to allow for a more granular, policy-relevant understanding of regional disparities, consistent with the approaches used by AlShatti et al. (22) and El-Deen et al. (20).

### 4.4 Policy implications

This study identified several areas that require policy intervention. First, developing a targeted rural healthcare infrastructure could reduce transportation costs and improve service accessibility. Second, insurance reform could enhance coverage for specialized services, as suggested by Alanazi and Benlaria (12). Current insurance frameworks cover only basic medical services, leaving families to pay out-of-pocket for essential therapies, averaging SAR 1,800 (USD 480) monthly. Third, establishing a centralized database for tracking healthcare costs and service utilization would facilitate evidence-based policy development, supporting Moynihan et al.’s (26) emphasis on a continuous service evaluation.

The analysis of service integration levels reveals specific opportunities for system-wide improvement. Based on Table 5, while medical care shows high integration (0.82), moderate integration levels of special education (0.70) and transportation services (0.65) suggest areas requiring focused intervention. These findings support the need for a comprehensive approach to service delivery, particularly considering the interconnected nature of the healthcare needs of children with DS.

Policy recommendations emerge from cost burden analysis. The data indicates that low-income families (<SAR 8,000/USD 2,133 monthly) spend up to 52.5% of their income on care-related expenses in urban areas and 48.8% in rural regions. This substantial financial burden suggests the need for income-based support mechanisms similar to those implemented in other healthcare systems. Costanzo and Magnuson (24) documented the effectiveness of tiered support systems that adjust assistance levels according to family income and specific care needs. The current government support of SAR 2,100 (USD 560) monthly covers only 28% of average care costs, substantially lower than the support levels in countries with comprehensive disability programs.

### 4.5 Service delivery patterns

Examination of healthcare access patterns reveals a complex relationship between service availability and utilization. Emergency services maintain relatively high access rates across regions (urban: 94%; rural: 89%), demonstrating that equitable service delivery is achievable within the current system. Nevertheless, the considerable differences in therapy access and educational support (17 and 19%, respectively) across regions imply the presence of structural problems that require targeted interventions.

International evidence suggests that telehealth and mobile therapy services can help to bridge these gaps. Countries with similar geographic challenges have successfully implemented hub-and-spoke models, reducing rural–urban disparities by 30–40% (6). Saudi Arabia’s Vision 2030 healthcare transformation program provides a framework for implementing such innovations through systematic reforms.

This forms the basis of longitudinal monitoring and evaluation, which are critical for addressing the underlying aspects of sustainability. Creating a national database for healthcare expenditure and service utilization could help establish policies based on real-time data. This approach is similar to the established monitoring systems in other countries, as explained by Shetty et al. (8) in their review of economic burden assessment methods.

### 4.6 Policy implications and recommendations

Our findings point to several policy implications that could help address the identified disparities in healthcare access and financial burden for families of children with DS in Saudi Arabia.

The significant transportation cost differences between urban and rural areas (*p* = 0.008, *d* = 0.38) suggest the need for targeted infrastructure development in underserved regions. Rural families spend proportionally more on transportation (17% vs. 13% of total costs, SAR 663/USD 177 vs. SAR 546/USD 146), indicating geographical barriers to specialized care. Policymakers should consider establishing regional therapy and specialized care centers in rural provinces to reduce travel burdens and associated costs. Additionally, transportation assistance programs can help bridge access gaps for families in remote areas.

The urban–rural insurance coverage disparity (86% vs. 77%, *p* = 0.012) indicates structural inequities that require policy attention. Current insurance frameworks are insufficient for families to manage DS care, particularly in rural regions. Insurance reform should focus on expanding coverage for developmental therapies and specialized services, which constitute significant portions of family expenditure (24 and 28%, respectively, totaling SAR 3,900/USD 1,040 monthly). The government can implement targeted insurance subsidies for rural families to address the documented coverage gap.

The moderate integration scores for special education (0.70) and transportation services (0.65) highlight opportunities for system improvements. Our findings suggest that developing a coordinated service delivery framework would benefit families navigating a complex care landscape. This could include:

- Creating a centralized referral system to connect primary healthcare with specialized services
- Implementing a standardized assessment protocol for children with DS
- Developing integrated case management to reduce the coordinative burden on families

With government support currently covering only 28% of the average care costs (SAR 2,100/USD 560 of SAR 7,500/USD 2,000 total), there is a clear need for enhanced financial assistance programs. The differential impact of the current support mechanisms between urban and rural areas (−2.2, *p* = 0.044) suggests an inequitable distribution of resources. Policymakers should consider implementing income-based sliding-scale subsidies that account for both financial needs and geographical locations. Targeted support for therapy services would address a significant cost driver (*β* = 0.450, *p* < 0.001), while improving developmental outcomes.

The challenges encountered in obtaining comprehensive national data on DS prevalence and services suggest the need for improved information systems. A national registry of children with DS will facilitate evidence-based planning and resource allocation. Such a system could track healthcare utilization, costs, and outcomes, providing valuable data for ongoing policy refinement.

These recommendations align with Saudi Arabia’s Vision 2030 healthcare transformation goals, and would directly address the empirical disparities documented in our study. Implementation would require coordinated efforts across healthcare, education, and social service sectors but could substantially improve the support ecosystem for families of children with DS.

### 4.7 Limitations and future directions

Several limitations should be considered when interpreting the findings of this study. The sampling approach, which focused primarily on urban centers with a comparatively smaller representation from rural areas, may have introduced a geographical selection bias that limited the generalizability of our results across all Saudi contexts. Self-reported financial and healthcare utilization data are inherently susceptible to recall bias and social desirability effects despite rigorous validation procedures. While our sample size (n = 220) exceeded the minimum requirements for statistical power, larger samples, particularly from rural regions, would enhance the robustness of the regional disparity analyses.

The absence of clinical severity measures for Down syndrome represents a significant limitation as international studies have demonstrated that severity levels can be considered. Children with DS and complex cardiac conditions, for example, may incur costs to 2–3 times higher than those with milder presentations. The cross-sectional design captures only a single time point in the dynamic healthcare environment of Saudi Arabia, precluding causal inferences about cost determinants. Our regression model emphasizes cost decomposition and access gaps rather than exploring interaction effects, as future research using multilevel or interaction models could explore how regional factors moderate the effects of specific predictors. However, this alternative modeling approach is beyond the scope of this study.

Future investigations might benefit from mixed-method approaches that incorporate qualitative elements, such as focus group discussions, to capture the experiential dimensions of caregiving burden and service navigation. Longitudinal studies tracking cost patterns over time would also enhance our understanding of how financial challenges evolve across different developmental stages in children with Down syndrome in Saudi Arabia. Additionally, cost-effectiveness analyses that compare different service delivery models, including telehealth interventions, could inform policy decisions regarding resource allocation and service expansion in underserved areas.

## 5 Conclusion

This study highlights the economic challenges faced by Saudi families seeking care for their children with DS. Our analysis demonstrates that families spend an average of SAR 7,500 (USD 2,000) monthly on DS-related care, representing 60% of the household income, which is substantially higher than the WHO threshold of 40% for catastrophic health expenditure. Our findings revealed significant disparities in healthcare costs and access between urban and rural regions, supported by statistical evidence of inequities across multiple domains. Specifically, rural families face a 17–19% disadvantage in accessing therapy and educational services (*p* < 0.001) while bearing proportionally higher transportation costs (17% vs. 13% of total expenses, *p* = 0.008). The results indicate a lack of coordination in financing healthcare delivery between the public and private sectors, underscoring the need to address issues stemming from the dual nature of the Saudi Arabian healthcare system and the regional imbalances that exacerbate this situation.

This study contributes to the literature in several ways. First, it provides the first comprehensive economic analysis of DS care costs in Saudi Arabia, filling a critical gap in the regional healthcare economics research. Second, it quantifies the differential impact of government support programs across urban and rural settings, revealing that the current support for SAR 2,100 (USD 560) monthly covers only 28% of care costs. Third, the Service Integration Index demonstrates that, while basic medical care achieves high integration (0.82), critical developmental services such as special education (0.70) and transportation (0.65) remain inadequately integrated.

Based on our statistical analysis, the most urgent policy needs include (1) addressing the significant urban–rural disparity in therapy access (17% gap, *p* = 0.003) and transportation costs (SAR 663/USD 177 vs. SAR 546/USD 146), which represent critical barriers for rural families; (2) reforming insurance coverage to bridge the documented gap between urban (86%) and rural regions (77%), with particular emphasis on covering developmental therapies currently averaging 1,800 (USD 480) monthly out-of-pocket; and (3) enhancing government support mechanisms, which currently cover only 28% of total care costs and exhibit uneven impact across urban and rural areas (−2.2 differential impact, *p* = 0.044).

The practical implications of this study underscore the importance of strengthening financial planning and support systems to alleviate these burdens. Effective strategies include improving public aid programs with a focus on regional equity, enhancing insurance coverage to minimize the substantial out-of-pocket expenses that consume 72% of the total care costs, and rectifying the regional imbalance in specialized services through targeted infrastructure development. Our findings align with Andersen’s Behavioral Model, demonstrating that enabling factors (income, insurance, and geographic location) exert a stronger influence on healthcare utilization in the Saudi context than in developed healthcare systems, suggesting that structural barriers require priority attention. Additionally, early diagnosis and intervention, increased awareness of financial issues, and financial preparedness have emerged as crucial factors in managing the costs of long-term care services within Saudi Arabia’s evolving healthcare system.

Future research should explore the qualitative aspects of caregiver burden and assess the effectiveness of policy interventions in reducing financial strain and improving access to services. Longitudinal studies that track cost trajectories across developmental stages and cost-effectiveness analyses of alternative service delivery models, including telehealth interventions, would provide valuable evidence for policy refinement. As Saudi Arabia continues its healthcare transformation under Vision 2030, addressing these evidence-based needs will be essential for improving the outcomes of children with DS and their families. The establishment of a national DS registry and implementation of income-based support mechanisms could reduce the financial burden on sustainable levels, while ensuring equitable access to essential services across all regions.

## Data Availability

The original contributions presented in the study are included in the article/Supplementary material, further inquiries can be directed to the corresponding author/s.
