# Supplementary material for: Balancing costs and care: a healthcare cost analysis for families of children with Down syndrome in Saudi Arabia
Source: Front Public Health. 2025 Nov 11;13:1651534. doi: 10.3389/fpubh.2025.1651534 (PMC12644096; doi:10.3389/fpubh.2025.1651534)
Supplement: Supplementary file 1 [file Data_Sheet_1.PDF]

# Questionnaire

## SECTION 1: DEMOGRAPHIC INFORMATION

1. Your relationship to child with Down syndrome: □ Mother □ Father □ Other: __________
2. Child's age: _____ years _____ months
3. Child's gender: □ Male □ Female
4. Total family size (all household members): _____ persons
5. Parent/Caregiver education (highest level completed):
    - Mother: □ No formal □ Primary □ Secondary □ University □ Graduate
    - Father: □ No formal □ Primary □ Secondary □ University □ Graduate
6. Your region: □ Riyadh □ Al-Jouf

## SECTION 2: FINANCIAL INFORMATION

7. Monthly household income (SAR): □ <5,000 □ 5,000-8,000 □ 8,001-12,000 □ 12,001-15,000 □ 15,001-20,000 □ >20,000
8. Additional monthly household expenses (excluding DS care) (SAR): __________
9. Government support received monthly for DS care (SAR): __________
10. Health insurance coverage: □ Yes (Coverage: ____%) □ No

## SECTION 3: CARE PROVISION

11. Average hours of daily care for child with DS: _____ hours per day

## SECTION 4: MONTHLY CARE COSTS (SAR)

*Please provide your average monthly out-of-pocket expenses:*

12. Medical Expenses (consultations, medications, tests, hospital fees): __________
13. Therapy Costs (speech, occupational, physical therapy): __________
14. Educational Costs (school fees, special education, materials): __________
15. Transportation Costs (travel to medical/therapy/school): __________
16. TOTAL Monthly Care Costs for DS care: __________

## SECTION 5: SERVICE ACCESS

17. Do you have access to the following services? (Yes/No)

| Service | Access |
|---|---|
| Medical care | □ Yes □ No |
| Therapy services | □ Yes □ No |
| Special education | □ Yes □ No |
| Emergency services | □ Yes □ No |
| Insurance coverage | □ Yes □ No |
| Specialized DS services | □ Yes □ No |

18. Primary healthcare facility type: □ Public/MOH □ Private □ Both
